# EUS-guided coloenterostomy for the management of afferent limb syndrome-induced pancreatitis in a patient with donor duodenojejunostomy

**DOI:** 10.1016/j.vgie.2025.01.003

**Published:** 2025-01-07

**Authors:** Abhishek Satishchandran, Charles Meade, Seth A. Waits, Meredith Barrett, Allison R. Schulman

**Affiliations:** 1 Division of Gastroenterology and Hepatology, University of Michigan, Ann Arbor, Michigan, USA; 2 Department of Surgery, University of Michigan, Ann Arbor, Michigan, USA

Afferent loop syndrome, typically associated with foregut surgeries such as partial gastrectomy with Billroth II or Roux-en-Y reconstruction, results from obstruction of the afferent limb, leading to bowel distention and blockage of the pancreaticobiliary tree. This can cause stagnant secretions, increasing the risk of pancreatitis and ascending cholangitis.^1^, ^2^, ^3^, ^4^

Here we present a case of a 52-year-old man with a history of type I diabetes complicated by end-stage renal disease who underwent simultaneous pancreas–kidney transplant with creation of an end-to-side duodenojejunostomy (Video 1, available online at www.videogie.org). The creation of a duodenal pancreatic jejunostomy is a standard surgical technique used during pancreatic transplantation where the donor duodenum and pancreas is anastomosed to a jejunal loop in the right lower quadrant where native ampullary anatomy can be found.

Seven years after his transplant, he developed unexplained recurrent acute pancreatitis of his donor pancreas, characterized by lipase elevation and radiographic evidence of acute interstitial pancreatitis by computed tomography imaging (Fig. 1). The native pancreas was atrophic but otherwise normal (Fig. 2). Imaging demonstrated a dilated donor duodenum with upstream small-bowel dilation (Fig. 3). On closer inspection, there was suspicion of a compressed or angulated efferent limb. This appearance was concerning for an afferent limb-like syndrome causing recurrent acute pancreatitis of the donor pancreas from progressive dilation of the donor duodenum (Fig. 4). No pancreatic ductal dilation was observed, possibly because of an intact ampulla, which may be less susceptible to dynamic pressure changes than surgical ductal anastomoses.

**Figure 1:**
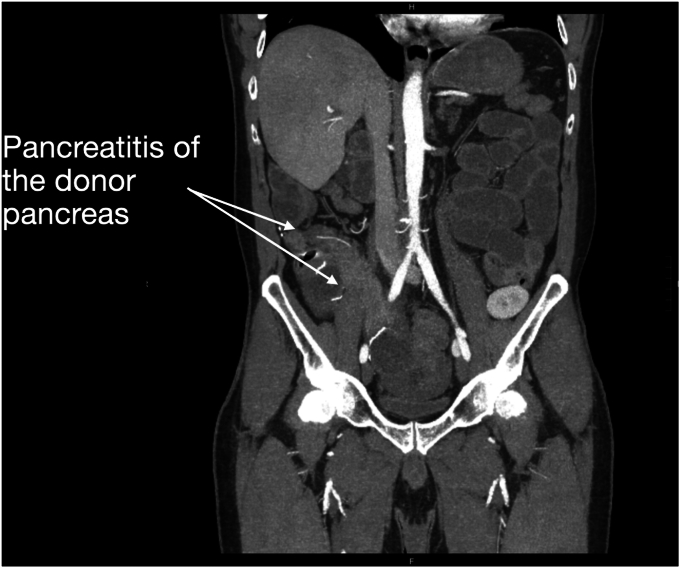
Intravenous contrast-enhanced computed tomography abdomen-pelvis acute interstitial pancreatitis of the donor pancreas with peripancreatic fat stranding. No pancreatic ductal dilation is seen.

**Figure 2:**
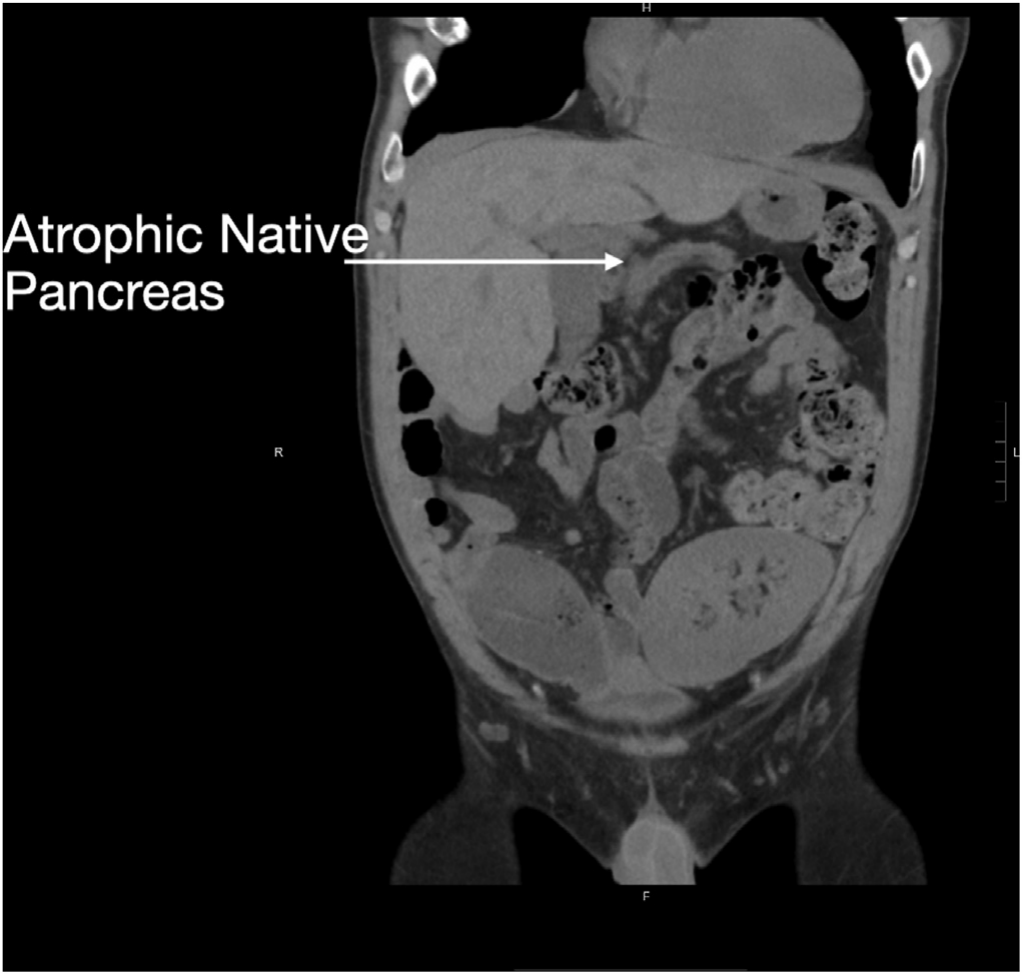
Intravenous contrast-enhanced computed tomography abdomen-pelvis demonstrating atrophic but otherwise normal-appearing native pancreas.

**Figure 3:**
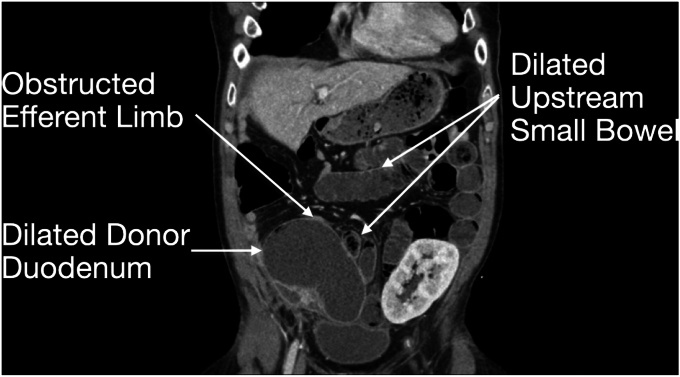
Intravenous contrast-enhanced computed tomography abdomen-pelvis demonstrating a dilated donor duodenum and upstream small bowel with a decompressed downstream efferent limb.

**Figure 4:**
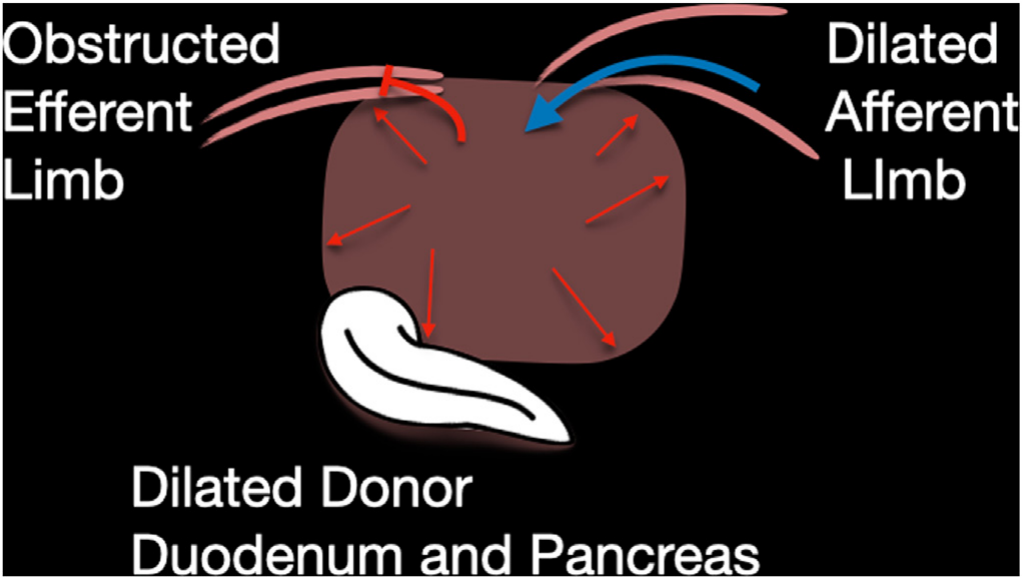
Schematic representation of the obstruction of the efferent limb.

Given his history of previous surgical interventions and transplant status, the decision was made to attempt a less-invasive approach to treatment of afferent-limb-like syndrome.^5^^,^^6^ Enteroscopy and percutaneous decompression were considered; however, given unknown limb length, complicated surgical history, and patient preference, these approaches were deferred. An EUS-guided cautery–enhanced 15-mm × 10-mm lumen-apposing metal stent (LAMS) was used to create a colo-enterostomy from the sigmoid colon to the dilated donor duodenum (Fig. 5). This would also allow for the evaluation of the efferent limb.

**Figure 5:**
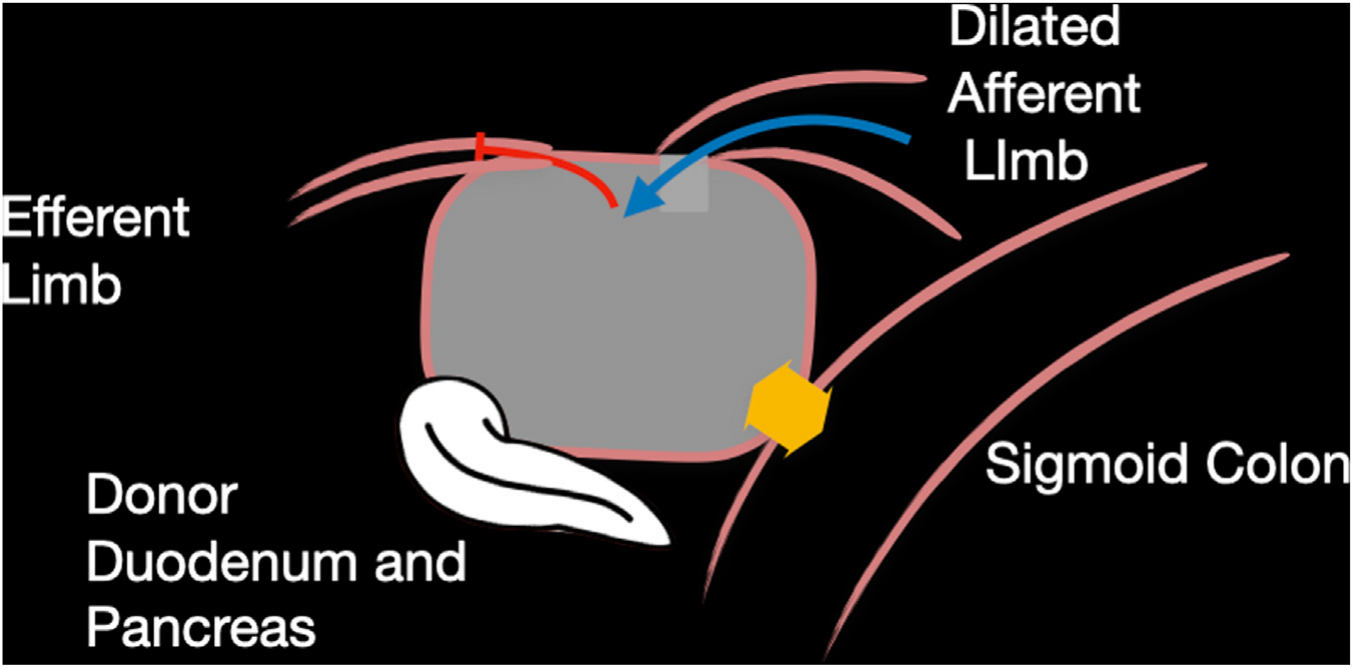
A schematic representation of the planned endoscopic ultrasound-guided placement of a lumen-apposing metal stent from the colon to the dilated donor duodenum, to allow for decompression and evaluation of the efferent anatomy.

After LAMS placement, the scope was advanced into the donor duodenum (Fig. 6). The ampulla was inspected and was normal in appearance. Acute angulation of the efferent limb was noted but not fully evaluated, given concern for LAMS dislodgement in the index procedure (Fig. 7).

**Figure 6:**
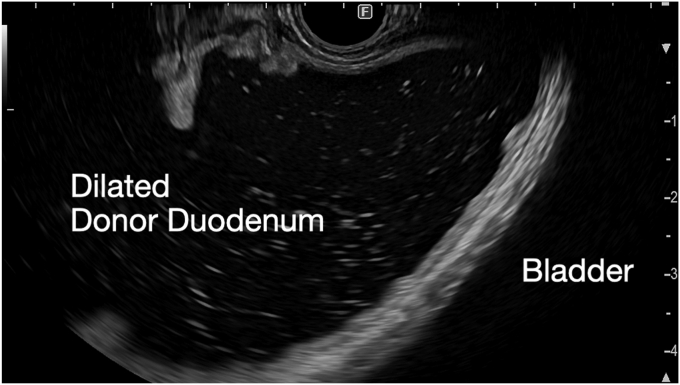
Representative trans-sigmoidal endosonographic image of the dilated donor duodenum adjacent to the bladder.

**Figure 7:**
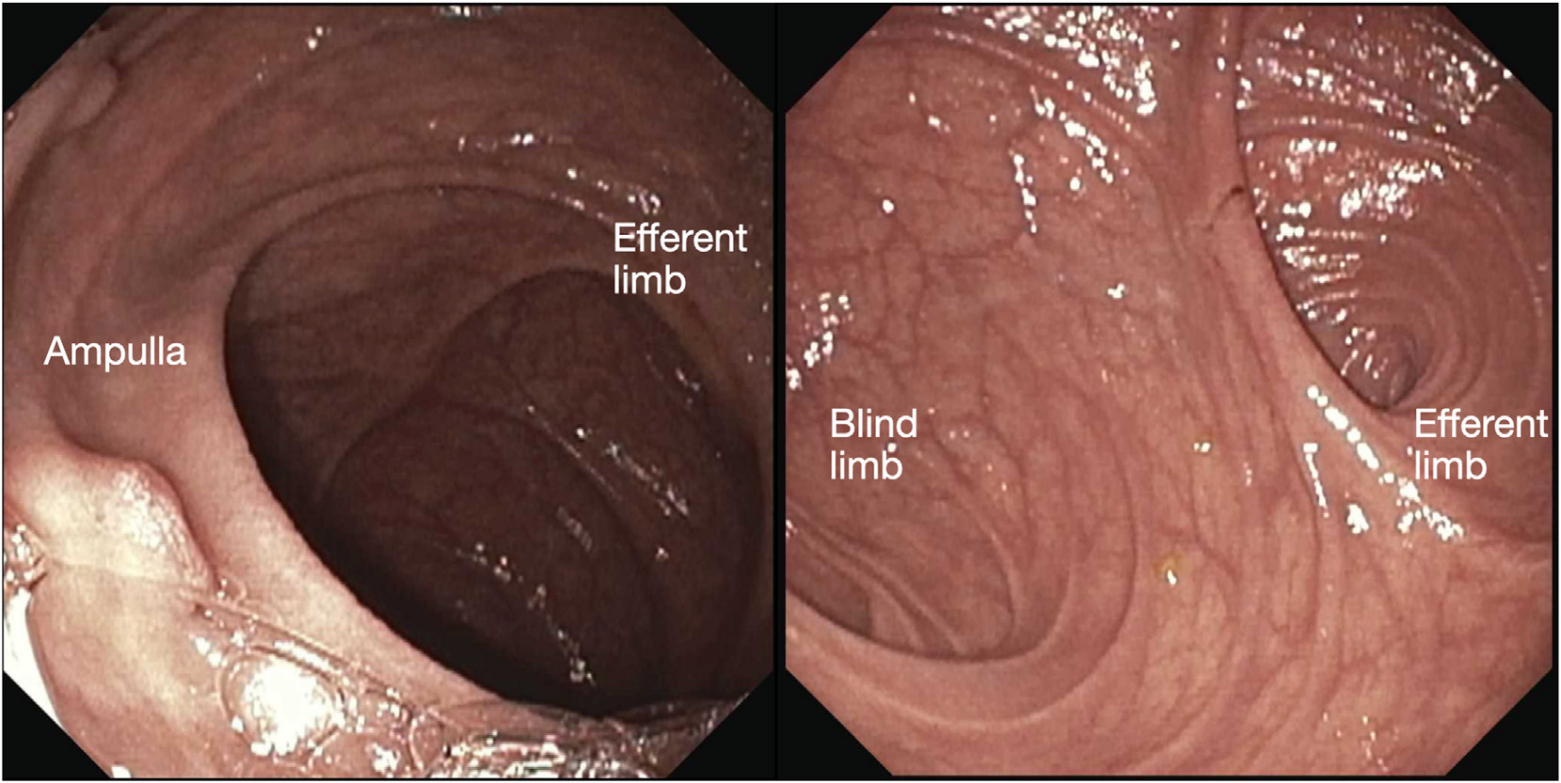
Endoscopic view of the dilated donor duodenum with the donor ampulla and the angulated takeoff of the efferent limb.

The patient reported significant diarrhea and was brought back to the endoscopy suite, likely attributable to iatrogenic short-gut syndrome 14 days after his initial procedure. Subsequent examination revealed that LAMS decompression of the donor duodenum improved efferent limb angulation (Fig. 8). Ultimately, the LAMS was removed, and plastic pigtail stents were placed to maintain the colo-enterostomy tract. This would allow for continued decompression of the donor duodenum to reduce the angulation of the efferent limb while also preventing recurrent pancreatitis. On follow-up, the patient had no further episodes of pancreatitis, abdominal pain, or emesis. Subsequent computed tomography scan demonstrated improved dilatation of the donor duodenum and resolution of pancreatitis.

**Figure 8:**
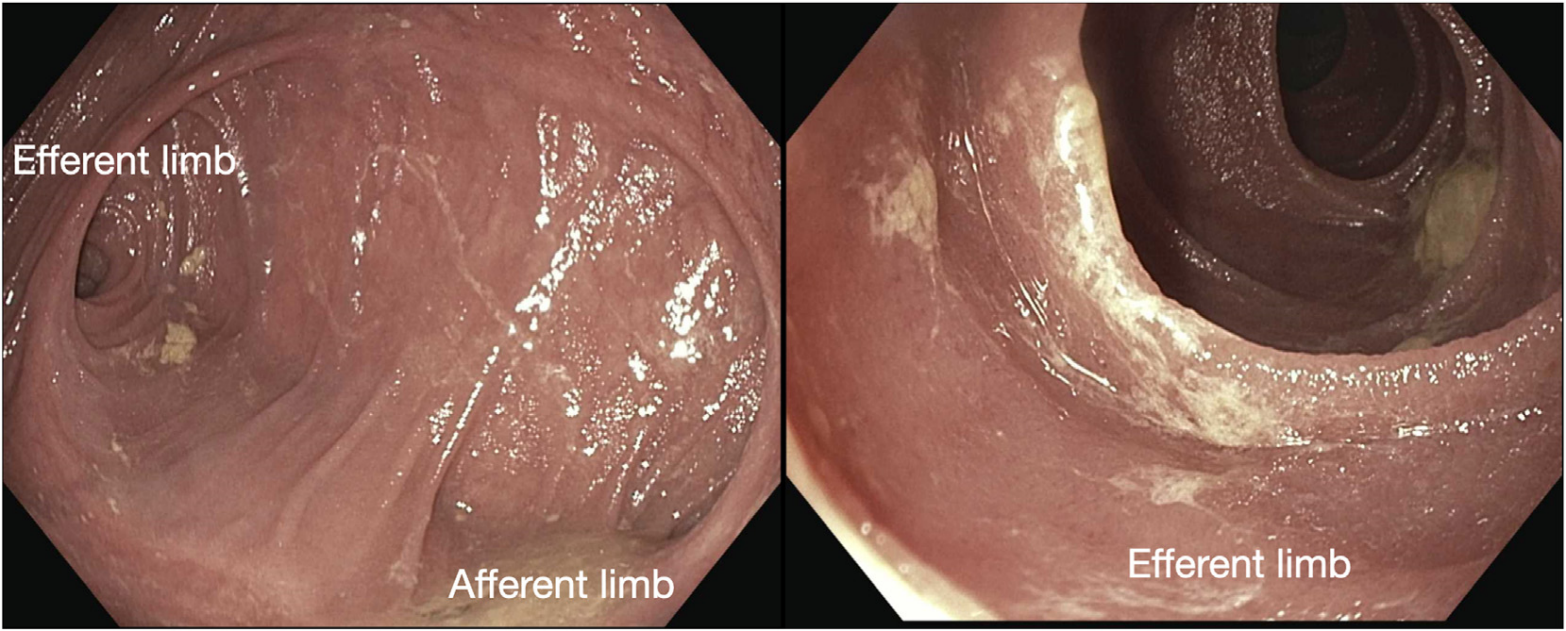
Endoscopic view of the dilated donor duodenum and decompressed efferent limb with improved angulation after lumen-apposing metal stent decompression.

## Conclusions

Our case demonstrates the effective use of EUS-guided LAMS placement to create a colo-enterostomy into a donor duodenum as an alternative treatment to surgical management of an afferent limb-like syndrome in suboptimal surgical candidates after multidisciplinary discussion.

## Patient Consent

The patient in this article has given informed consent to publication of the case details.

## Disclosure

Dr Schulman is a consultant for Apollo Endosurgery, Boston Scientific, Fractyl, Micro-Tech, and Olympus; is on the advisory board for Apollo Endosurgery; and receives research/grant support from GI Dynamics and Fractyl. The other authors disclosed no financial relationships.

## Supplementary data

Video 1 Video presentation of an EUS-guided LAMS placement to create a coloenterotostmy into a donor duodenum as treatment of an afferent limb-like syndrome in a patient with a pancreas transplant.
